# Novel docetaxel-loaded nanoparticles based on poly(lactide-co-caprolactone) and poly(lactide-co-glycolide-co-caprolactone) for prostate cancer treatment: formulation, characterization, and cytotoxicity studies

**DOI:** 10.1186/1556-276X-6-260

**Published:** 2011-03-28

**Authors:** Vanna Sanna, Anna Maria Roggio, Anna Maria Posadino, Annalisa Cossu, Salvatore Marceddu, Alberto Mariani, Valeria Alzari, Sergio Uzzau, Gianfranco Pintus, Mario Sechi

**Affiliations:** 1 Porto Conte Ricerche, Località Tramariglio, Alghero, Sassari 07041, Italy; 2 Department of Biomedical Sciences, Centre of Excellence for Biotechnology Development and Biodiversity Research, University of Sassari, Viale San Pietro 43/B, Sassari 07100, Italy; 3 Istituto di Scienze delle Produzioni Alimentari (ISPA), CNR, Via dei Mille 48, Sassari 07100, Italy; 4 Department of Chemistry and local INSTM unit, University of Sassari, Via Vienna 2, Sassari 07100, Italy; 5 Dipartimento di Scienze del Farmaco, University of Sassari, Via Muroni 23/A, Sassari 07100, Italy

## Abstract

Docetaxel (Dtx) chemotherapy is the optional treatment in patients with hormone-refractory metastatic prostate cancer, and Dtx-loaded polymeric nanoparticles (NPs) have the potential to induce durable clinical responses. However, alternative formulations are needed to overcome the serious side effects, also due to the adjuvant used, and to improve the clinical efficacy of the drug.

In the present study, two novel biodegradable *block*-copolymers, poly(lactide-co-caprolactone) (PLA-PCL) and poly(lactide-co-caprolactone-co-glycolide) (PLGA-PCL), were explored for the formulation of Dtx-loaded NPs and compared with PLA- and PLGA-NPs. The nanosystems were prepared by an original nanoprecipitation method, using Pluronic F-127 as surfactant agent, and were characterized in terms of morphology, size distribution, encapsulation efficiency, crystalline structure, and *in vitro *release. To evaluate the potential anticancer efficacy of a nanoparticulate system, *in vitro *cytotoxicity studies on human prostate cancer cell line (PC3) were carried out. NPs were found to be of spherical shape with an average diameter in the range of 100 to 200 nm and a unimodal particle size distribution. Dtx was incorporated into the PLGA-PCL NPs with higher (*p *< 0.05) encapsulation efficiency than that of other polymers. Differential scanning calorimetry suggested that Dtx was molecularly dispersed in the polymeric matrices. *In vitro *drug release study showed that release profiles of Dtx varied on the bases of characteristics of polymers used for formulation. PLA-PCL and PLGA-PCL drug loaded NPs shared an overlapping release profiles, and are able to release about 90% of drug within 6 h, when compared with PLA- and PLGA-NPs. Moreover, cytotoxicity studies demonstrated advantages of the Dtx-loaded PLGA-PCL NPs over pure Dtx in both time- and concentration-dependent manner. In particular, an increase of 20% of PC3 growth inhibition was determined by PLGA-PCL NPs with respect to free drug after 72 h incubation and at all tested Dtx concentration. In summary, PLGA-PCL copolymer may be considered as an attractive and promising polymeric material for the formulation of Dtx NPs as delivery system for prostate cancer treatment, and can also be pursued as a validated system in a more large context.

## Introduction

Prostate cancer (PCa) is the most common male malignancy and the second leading cause of cancer death in Western industrialized countries [1]. The PCa progression does not only mean distant metastases but also development of hormone independence of its cells [2].

Docetaxel (Dtx)-based regimen is recommended as optional treatment in patients with hormone-refractory metastatic PCa, showing effective on locally advanced PCa and increasing overall survival [3]. Despite the recently reported promising outcome of Dtx, this drug is associated with systemic toxicity that limits the dose and duration of therapy, particularly in elderly patients. Adverse effects of Dtx treatment include hypersensitivity reactions, bone marrow suppression, cutaneous reactions, fluid retention, peripheral neuropathy, alopecia, cardiac disorders, and tiredness [4]. Moreover, to facilitate its clinical use, the Dtx poor water solubility requires a specific solvent system, such as ethanol/Tween 80 solution, which causes hypersensitivity reaction, reduced uptake by tumor tissue and increased exposure to other body compartments [5].

Therefore, alternative Dtx formulations are needed and nanoparticles (NPs)-based drug delivery systems are of special interest in addressing the major side effects of anticancer drug.

Recently, a number of polymers have been investigated for formulating biodegradable Dtx-loaded NPs for cancer chemotherapy. The most widely used class of biocompatible and biodegradable polymers, approved by Food and Drug Administration (FDA), is that of aliphatic polyesters, including poly(lactic acid) (PLA), poly(glycolic acid) (PLGA), and their copolymers [6].

However, these polymers are characterized by a high hydrophobicity and slow degradation rate that can represent in some cases a disadvantage for their use as drug carriers [7].

In the family of polyesters, polycaprolactone (PCL) shows an excellent biocompatibility and rapid degradability, good miscibility with a variety of polymers and great permeability, which make it a suitable candidate for drug carrier [8].

The methods for the preparation of NPs from the preformed polymers include nanoprecipitation, emulsification/solvent evaporation, emulsification/solvent diffusion, and salting out [9]. As for the nanoprecipitation, the particle formation is based on precipitation and subsequent solidification of the polymer at the interface between a solvent and a non-solvent [10]. Usually, surfactants or stabilizers are included in the process to modify the size and the surface properties, and to ensure the stability of the NP dispersion [9]. The most popular stabilizer for the production of PLGA-based NPs is poly(vinyl alcohol) (PVA). However, it has been found that residual PVA, which is very difficult to remove from the particles' surface, causes relatively lower cellular uptake, thus resulting not satisfactorily biocompatible for the human body [11]. Therefore, other surfactants could be considered as valuable alternatives to PVA. Poloxamer, a block copolymer of poly(ethylene oxide)-poly(propylene oxide)-poly(ethylene oxide), commercially known as Pluronic, is another promising surface active agent approved by FDA for clinical use [12]. Additionally, several evidence demonstrated that poloxamers interact with multidrug-resistant tumors, resulting in a drastic sensitization of these tumors with respect to various anticancer agents [13,14].

The present work is aimed at investigating the influence of two previously unexplored biodegradable *block*-copolymers, such as poly(D,L-lactide-co-caprolactone) (PLA-PCL) and poly(L-lactide-co-caprolactone-co-glycolide) (PLGA-PCL), on the physicochemical and pharmaceutical properties of novel Dtx-loaded NPs, using PLA- and PLGA-NPs as comparison. The designed nanosystems were obtained by a modified nanoprecipitation technique, using Pluronic F-127 instead of PVA as stabilizing agent. The NPs were characterized in terms of morphology, encapsulation efficiency and crystalline structure, and *in vitro *release kinetics. Moreover, the antiproliferative efficacy of the most promising nanoparticulate prototype was assessed by *in vitro *cytotoxicity assay toward a human prostate cancer cell line (PC3).

## Materials and methods

### Materials

Poly(D,L-lactide) (PLA) (inherent viscosity 0.55 to 0.75 dl/g in chloroform), poly(D,L-lactide-co-glycolide) (PLGA) (lactide:glycolide 50:50 molar ratio, inherent viscosity 0.55 to 0.75 dl/g in hexafluoroisopropanol) with esters end groups were purchased from Lactel Absorbable Polymers (Pelham, AL, USA). Poly(L-lactide-co-caprolactone-co-glycolide) (PLGA-PCL) (L-lactide:glycolide:caprolactone 70:10:20 molar ratio, inherent viscosity 1.30 dl/g, in chloroform) was obtained from Sigma-Aldrich (Steinheim, Germany). Poly(L-lactide-co-caprolactone) (PLA-PCL) (lactide:caprolactone 70:30 molar ratio) was kindly provided from Purac Biomaterials (Gorinchen, Netherlands). Pluronic^® ^F-127 was obtained from Sigma-Aldrich (Steinheim, Germany). Docetaxel (purity 99%) was supplied by ChemieTek (Indianapolis, IN, USA). All other chemicals were analytical grade and were used without further purification.

### Formulation of nanoparticles

Drug-loaded NPs were prepared by a novel nanoprecipitation method. Briefly, about 100 mg of polymer and drug (1%, w/w) were dissolved in 6 ml of acetonitrile. The organic solution was then added dropwise under stirring to an aqueous solution of Pluronic F-127 (1% w/v, 30 ml). The milky colloidal suspension was evaporated at room temperature to remove the organic solvent. The suspension was centrifuged at 13,000 rpm for 10 min to separate the NPs from the unencapsulated drug and residual Pluronic. The pellet was washed thrice with ultrapure water. Drug-free NPs were produced in a similar manner. The obtained NP suspension was used immediately for assay or lyophilized for storage at -50°C.

### Characterization of NPs

#### Particle size and size distribution

Mean diameter and size distribution of the NPs were measured by photon correlation spectroscopy with Coulter N4 plus particle size analyzer (Beckman Coulter, Fullerton, CA, USA). The samples were diluted with deionized water and sonicated for 5 min before measurement. Each analysis was carried out in triplicate.

#### Surface morphology

The morphological examination of NPs was performed by scanning electron microscopy (SEM) (model DSM 962; Carl Zeiss Inc., Oberkochen, German). A drop of NPs suspension was placed on a glass cover slide and dried under vacuum for 12 h. After that, the slides were mounted on aluminum stub and the samples were then analyzed at 20 kV acceleration voltage after gold sputtering, under an argon atmosphere.

#### Encapsulation efficiency and yield of production

To determine the drug loading, an aliquot of NPs (1.0 mg) was dissolved in 0.25 ml of acetonitrile/water (50/50, v/v) solution. Samples were centrifuged and the drug content in the supernatant was analyzed by HPLC using the method previously reported [15]. The equipment consisted of an HPLC system including an autosampler and a diode array detector (HP 1200, Agilent Technologies, Santa Clara, CA, USA). The chromatographic separation was performed at room temperature using a Jupiter C18 column (250 mm × 2.0 mm, 5 μm pore size, Phenomenex) with a flow rate of 0.2 ml/min. Twenty microlitres of samples or calibration standards were injected into the column and eluted with a solution of acetonitrile/water solution (50/50, v/v). Detection was carried out by monitoring the absorbance signals at 227 nm. The elution period was 25 min and the retention time of Dtx was about 9 min. HPLC was calibrated with standard solutions of 5 to 50 μg/ml of Dtx (correlation coefficient of *R*^2 ^= 0.9999).

The drug encapsulation efficiency (EE%) was calculated as the ratio between the amount of Dtx encapsulated in NPs and the weight of drug used for NP preparation.

The lyophilized NPs from each formulation were weighed and the respective percentage yield of production was calculated as the ratio between the amount of NPs weight obtained and the total weight of solid materials used for the preparations.

### Differential scanning calorimetry (DSC)

DSC scans of empty and drug-loaded NPs were performed on a DSC Q100 V 9.0 calorimeter (TA Instrument, New Castle, USA). Indium was used to calibrate the instrument. The thermograms of samples were obtained at a scanning rate of 10°C/min in 30 to 200°C temperature range and performed under an Ar purge (50 ml/min). The thermal measurements were carried out on pure Dtx, on freeze-dried empty and drug-loaded NPs.

### *In vitro *drug release

For the *in vitro *release studies, about 10 mg of Dtx-NPs were suspended in 2.0 ml of release medium (phosphate buffer solution of pH 7.4 containing 0.1% w/v Tween 80). The tubes were then incubated at 37°C and shaken horizontally at 200 rpm (Thermomixer HLC BioTech, Bovenden, Germany). At predetermined time intervals, the tubes were centrifuged at 13,000 rpm for 10 min and 1.0 ml aliquots of medium were taken from supernatant and replaced with fresh solution. The collected samples were extracted with 0.2 ml dichloromethane, evaporated by nitrogen stream, and reconstituted in 100 μl mobile phase.

The concentration of Dtx released from the NPs was determined by HPLC assay as described above and corrected for the dilution due to the addition of fresh medium.

### *In vitro *cytotoxicity assay

With respect to particle size, encapsulation efficiency, and yield of production results, the PLGA-PCL Dtx-loaded NPs were selected for the *in vitro *cytotoxicity studies. A batch of empty PLGA-PCL NPs was used as comparison.

Cancer cell viability of the drug-loaded NPs was evaluated by MTT assay. Hundred microliters of PC3 cells were seeded in 96-well plates (Costar, IL, USA) at the density of 5,000 viable cells/well and allowed to adhere for 24 h.

The cells were incubated with free Dtx, Dtx-loaded NP suspension at 0.05, 0.5, 5.0 μg/ml equivalent Dtx concentrations, and empty NPs with the same NP concentrations, for 24, 48, and 72 h, respectively. The selected concentrations were in the range which corresponds to plasma levels of the drug achievable in humans [16]. At designated time intervals, the medium was removed, and the wells were washed twice with PBS. Ten microliters of 3-(4,5-dimethyl-thiazol-2-yl)-2,5-diphenyltetrazolium bromide (MTT) solution were added to each well of the plate and incubated for another 1 to 4 h. The absorbance was measured at 560 nm using a microplate reader. Cell viability was calculated by the following equation:

where Abs_sample _is the absorbance of the cells incubated with the NP suspension, and Abs_control _is the absorbance of the cells incubated with the culture medium only (positive control). All experiments were repeated thrice.

### Statistical methodology

The data are expressed as mean ± standard deviation (S.D.). The significance of differences was assessed by one-way analysis of variance (ANOVA) (Origin^®^, version 7.0 SR0, OriginLab Corporation, Northampton, MA, USA) and considered significant when *p *< 0.05.

## Results and discussion

### Characterization of NPs

#### Size, drug entrapment efficiency, and yields of production

The nanoprecipitation method described here appeared to be a suitable technique to formulate Dtx-loaded NPs based on PLA and PLGA and their poly-caprolactone copolymers. The preparation was carried out by using a polymer concentration in organic phase of 3.3 mg/ml and a solvent:Pluronic^® ^F-127 solution ratio of 1:5. Acetonitrile was selected as organic phase for the capability to solubilize the polymers and drug and its good water miscibility, that allows a fine polymer dispersion into water.

Moreover, although the most commonly used PVA emulsifier can make uniform particles with small size, it has been recently demonstrated that it can form an interconnected network with the PLGA polymer at the interface that would compromise the good redispersibility of NPs [11]. Additionally, the residual PVA associated with the surface of NPs would influence their physical properties and cellular uptake. Therefore, due to the above reasons, PVA was conveniently replaced with Pluronic in the current study.

The prepared NPs were characterized in terms of particle size and size distribution, morphology, encapsulation efficiency, and yield of production. The effect of different polymers on the characteristics of NPs is summarized in Table 1.

**Table 1:** The effect of the different type of polymers on characteristic of NPs.

Polymeric NPs	Size (nm)	PDI	EE (%)	YP (%)
PLA	146.3 ± 23.5	0.198 ± 0.03	37.8 ± 3.0	51.9 ± 1.4
PLGA	106.7 ± 13.5	0.108 ± 0.04	35.2 ± 2.6	68.2 ± 2.7
PLA-PCL	194.8 ± 31.2	0.308 ± 0.08	19.7 ± 1.4	36.8 ± 1.6
PLGA-PCL	114.8 ± 15.4	0.258 ± 0.07	47.2 ± 3.8	83.9 ± 3.3

Data are shown as mean ± S.D., obtained from three formulations. PDI, polydispersity index; EE, encapsulation efficiency; YP, yield of production

**Figure 1:**
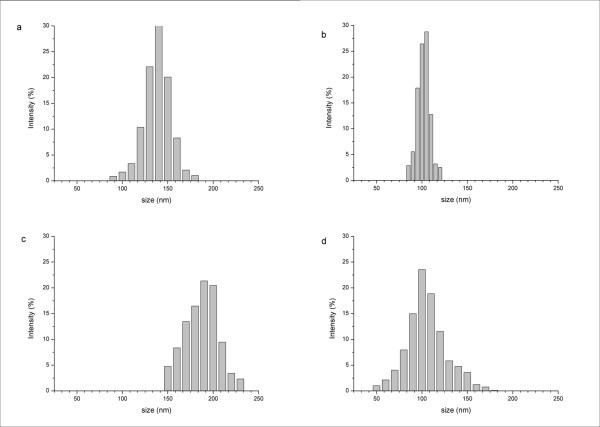
**Particles size distribution of PLA-Dtx (a), PLGA-Dtx (b), PLA-PCL-Dtx, (c) and PLGA-PCL-Dtx-loaded NPs (d)**.

All NP batches showed a particle diameter ranging from 100 to 200 nm, suitable to obtain an effective intracellular uptake [17]. NPs formulated with PLA-PCL polymer showed a significantly larger (*p *< 0.05) mean diameter (195 nm) than that of the PLGA- and PLGA-PCL-Dtx-loaded NPs (107 and 115 nm, respectively). As depicted in Figure 1, in all cases, the NP dispersions exhibit a unimodal particle size distribution, as also confirmed by the obtained low polydispersity indexes (PDI < 0.31), typical of monodispersed systems. This suggests that the presence of Pluronic F127 can promote the formation of very homogeneous NP dispersions.

As far as the drug entrapment efficiency (EE) is concerned, from drug content analysis no significant differences were found for the batches based on PLA and PLGA polymers (38 and 35%, respectively). On the other hand, relevant differences are obtained for NPs formulated with both copolymers containing PCL. The PLGA-PCL NPs exhibited significantly (*p *< 0.05) higher drug encapsulation efficiency (47%) compared with other formulations.

This finding can be ascribed to the presence of three polymers, PLA, PLGA, and PCL, which are able to generate a polymer network characterized by a greatly irregular and disordered crystalline structure, which may consequently promote the accommodation of the drug molecules.

Conversely, the lower percentages of encapsulation efficiency and NP yield obtained with PLA-PCL copolymer might be explained with a partial and fast precipitation of polymer that occurs after addition of organic phase to surfactant solution. Yields of production ranged between 52 and 84%, with the highest values for NPs based on PLGA-PCL copolymers.

Furthermore, the size and particle size distribution of unloaded NPs were similar to the corresponding loaded batches, thus indicating that the presence of the drug did not influence these characteristics (data not shown).

### Surface morphology

**Figure 2:**
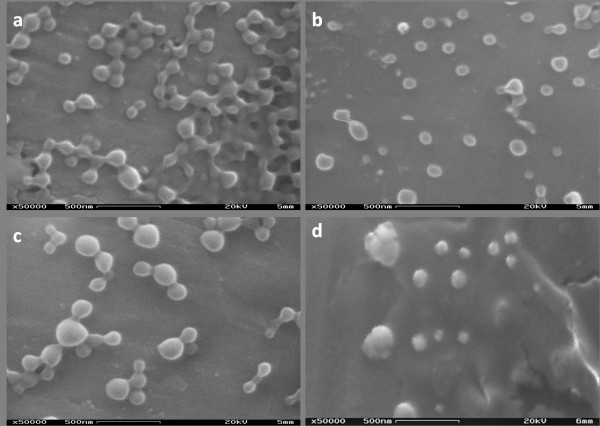
**Scanning electron microscopic images of PLA-Dtx (a), PLGA-Dtx (b), PLA-PCL-Dtx (c), and PLGA-PCL-Dtx-loaded NPs (d)**.

The morphology of NPs was investigated by SEM analysis. As displayed in Figure 2, all batches of NPs showed a spherical shape with a smooth surface, without any particle aggregation.

The partial fusion detected in some NP samples takes place during the analysis, where many NPs congregate together. Due to presence of the drug, no difference in the morphological properties of NPs drug was observed due to the presence of the drug.

### Differential scanning calorimetry (DSC)

**Figure 3:**
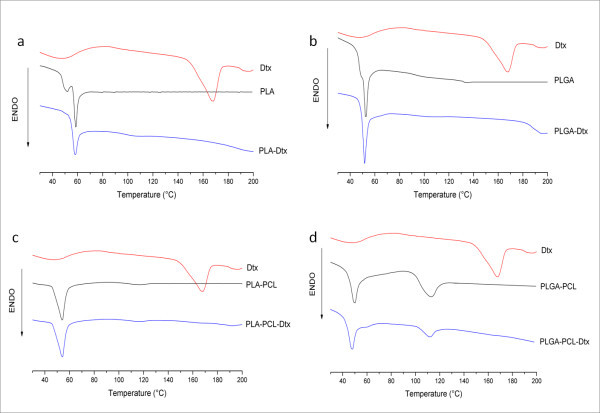
**DSC thermograms of pure Dtx compared with unloaded and loaded PLA (a), PLGA (b), PLA-PCL (c), and PLGA-PCL NPs (d)**.

The physical state of Dtx inside the NPs was characterized by analysis of the DSC curves. The pure drug shows a characteristic endothermic peak that corresponds to melting at 168°C, indicating a crystalline nature (Figure 3). The comparison between DSC thermograms of drug with unloaded and loaded NPs formulated with different polymers showed similar behaviors.

In all cases, the DSC scans revealed that Dtx melting peak totally disappeared in the calorimetric curve of loaded NPs, thus evidencing the absence of crystalline drug in the samples. These results suggested that the nanoencapsulation inhibited the crystallization of Dtx during NPs formation. Thus, it can be assumed that Dtx was present in the NPs either in an amorphous or disordered crystalline phase or in the solid solution state [6].

Meanwhile, the loaded systems exhibited the same endothermic events (at 58°C for PLA, 52°C for PLGA, 54°C for PLA-PCL, and 50 and 113°C for PLGA-PCL) of the corresponding unloaded NPs, referring to melting temperature (Tm) of polymers (Figure 3a to 3d), suggesting that the nanoencapsulation procedure did not affect the polymer structure.

### *In vitro *drug release

**Figure 4:**
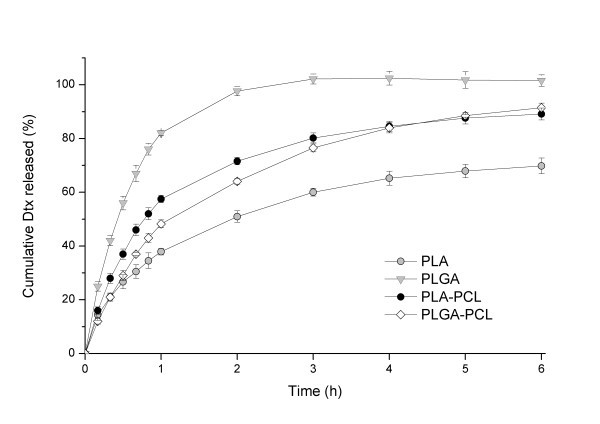
***In vitro *release profiles of Dtx-loaded PLA, PLGA, PLA-PCL, and PLGA-PCL NPs**.

The cumulative percentages of Dtx released from NPs based on different polymers as a function of time are reported in Figure 4. All formulations are characterized by similar release profiles, but the release rate is strongly affected by the properties of the polymer matrix. In particular, PLA-based NPs are characterized by the slower release (only about 60% of Dtx is released within 6 h) that can be attributed to the stronger hydrophobic interaction between hydrophobic domain of PLA and drug. Due to the presence of the hydrophilic glycolide units into the PLA polymer (50:50 molar ratio), in the case of NPs formulated with PLGA a completed dissolution of Dtx is achieved within the first 2 hof the test. In fact, the higher hydrophilicity of copolymer improved the water permeability into NPs with a more rapid drug diffusion as well as a faster degradation of the polymer [8]. As for PCL copolymers, the diffusion rate of drug from PLA-PCL NPs is higher at the first 2 h and levelled off after 3 h of the test, thus resulting almost superimposed to those of PLGA-PCL formulations. These results can be related to the similar composition of PLA-PCL and PLGA-PCL copolymers containing a lactide:caprolactone 70:30 and lactide:glycolide:caprolactone 70:10:20 molar ratio, respectively. Moreover, with respect to PLGA, a significant retard on dissolution rate of Dtx from PLGA-PCL NPs alone was obtained, whereas an opposite effect is observed in the case of PLA-PCL copolymer compared to PLA-NPs. Furthermore, with respect to PLA homopolymer, the more hydrophilic block of caprolactone of PLA-PCL copolymer-based NPs leads to a significant improvement of Dtx released (85%, during the 6 h). A complete release of Dtx is obtained for all samples after 24 h of the test.

In order to investigate the kinetic modeling of drug release from NPs, the dissolution profiles were fitted to zero order (*Q *= *k*_0_·*t*); first order (ln (100 - *Q*) = ln *Q*_0 _- *k*_1_·t), Higuchi (*Q *= *k*_H_·*t*^1/2^), and Korsmeyer-Peppas models [18].

Table 2 shows the correlation coefficient (*r*^2^) used as an indicator of the best fitting of the models considered for different Dtx-NPs batches. The *r*^2 ^values for first order kinetics of different polymeric NPs (ranging between 0.8830 and 0.9921) were greater than that of Zero order and corresponded to that of the Higuchi's square root of time (ranged between 0.8182 and 0.9813). Besides, to understand the drug release mechanism, the first 60% drug release data was fitted to Korsmeyer-Peppas exponential model *M*_t_/*M*_∞ _= *Kt^n ^*, where *M*_t_/*M*_∞ _is fraction of drug released after time '*t*' and '*K*' is kinetic constant and '*n*' is release exponent, which characterizes the different drug release mechanism [19]. Based on various mathematical models, the magnitude of the release exponent '*n*' indicates the release mechanism (Fickian diffusion, case II transport, or anomalous transport). The limits considered were *n *≤ 0.43 (for a classical Fickian diffusion-controlled drug release) and *n *= 0.85 (indicates a case II relaxational release transport; non-Fickian, zero-order release). Values of n between 0.43 and 0.85 can be regarded as an indicator of both phenomena (drug diffusion in the hydrated matrix and the polymer relaxation) usually called anomalous transport [19]. In all formulations, *n *values ranged from 0.5141 to 0.7842, suggesting an anomalous or non-Fickian diffusion, which is related to combination of both diffusion of the drug and dissolution of the polymer.

**Table 2:** Correlation coefficients (*r*^2^) and release exponent (*n*) of kinetic data analysis of Dtx release from different polymeric NPs.

Polymeric NPs	Zero order *r*^2^	First order *r*^2^	Higuchi *r*^2^	Koresmeyer-Peppas	
				*r*^2^	*n*
PLA	0.8875	0.9481	0.9813	0.9962	0.5141
PLGA	0.6295	0.8830	0.8182	0.9942	0.6706
PLA-PCL	0.7997	0.9503	0.9280	0.9956	0.7166
PLGA-PCL	0.8797	0.9921	0.9747	0.9988	0.7842

### *In vitro *cytotoxicity assay

The *in vitro *cytotoxicity of Dtx both as free drug and loaded into PLGA-PCL NPs, at the same drug equivalent concentration of 0.05, 0.5, and 5.0 μg/ml, was evaluated by the MTT assay using PC3 as model prostate cancer cell line. Untreated PC3 cells as well as cells treated with empty PLGA-PCL NPs, with the same polymer content of that of the drug-loaded nanosystems, were used as comparison.

**Figure 5:**
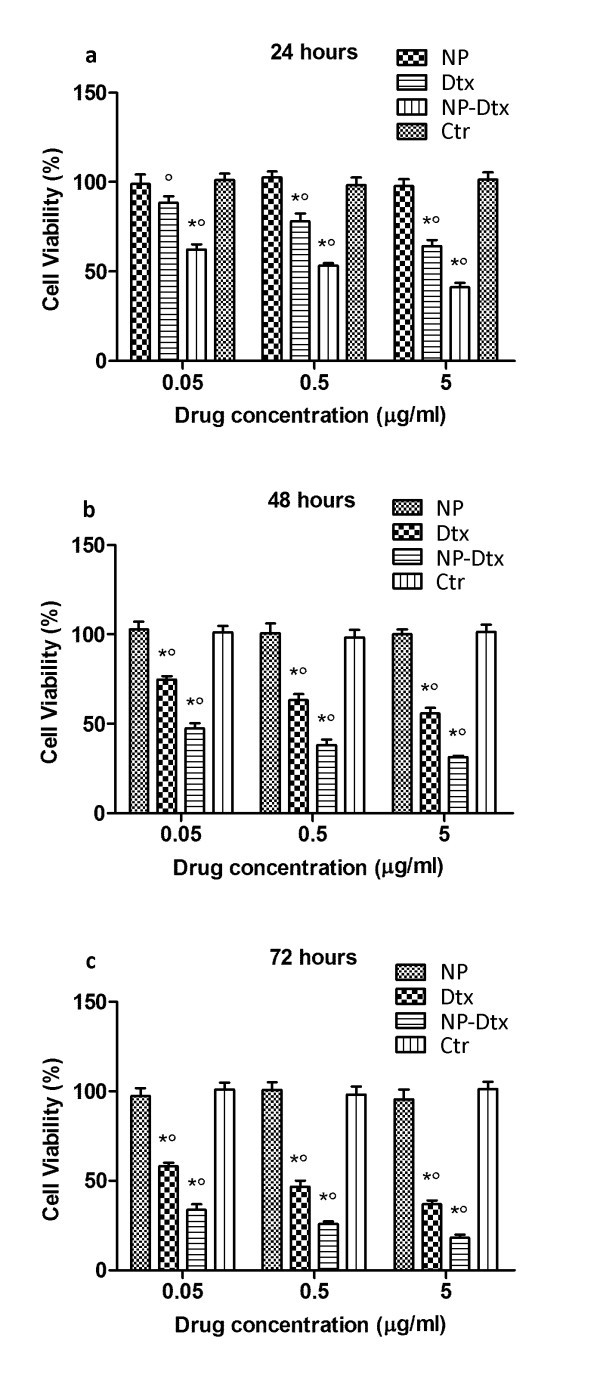
**Viability of PC3 cells cultured with drug-free nanoparticles (NP), docetaxel-loaded PLGA-PCL NPs (NP-Dtx) after 24 h (a), 48 h (b) and 72 h (c), in comparison with that of pure drug (Dtx) at the same dose (*n *= 3)**. Asterisks, significantly different from control; circle, significantly different from each other.

Figure 5 shows the viability of PC3 cancer cells, cultured with unloaded PLGA-PCL NPs, and Dtx-loaded PLGA-PCL NPs, after incubation for 24 h (**a**), 48 (**b**), and 72 h (**c**), in comparison with that of pure drug at 0.05, 0.5, and 5.0 μg/ml equivalent Dtx concentrations.

Since no significant influence on cell viability was detected, drug-free NPs appeared nearly non-toxic to PC3 cells, even at a high NP concentration, thus confirming the good biocompatibility of PLGA-PCL copolymer [20].

On the whole, the results clearly demonstrated that Dtx formulated in PLGA-PCL NPs resulted more effective against cancer cells than free Dtx. Both the dose exposure and the incubation time played a major role in the cell toxicity of Dtx.

In particular, after 24 h incubation, the PC3 cell viability was decreased to about 65, 55, and 40% for PLGA-PCL NPs at 0.05, 0.5, and 5.0 μg/ml drug concentration, respectively, corresponding to an increase in cytotoxicity of 25% (*p *< 0.05) compared with that of free Dtx.

After 48 h incubation, the cytotoxicity was increased about 55, 60, and 70% for the PLGA-PCL-Dtx NPs and 25, 35, and 45% for free drug, respectively.

The more marked inhibition of cell growth was obtained for longer incubation period (72 h); at all tested Dtx concentration, PLGA-PCL NPs determined a significant (*p *< 0.05) increased of PC3 growth inhibition (about 20%) with respect to free drug. With an approximately 85% cell viability reduction, the strongest cytotoxic effect was achieved with NPs at 5.0 μg/ml Dtx concentration.

This finding is related with the mechanism of action of Dtx, which requires cell division to operate. In fact, for longer incubation periods, a larger number of cells enter G2 and M cell cycle phases, where Dtx selectively works [21].

According to literature data, the higher cytotoxicity of the drug formulated into NPs can be attributed to the combination of different but not exclusive mechanisms. In fact, the NPs were adsorbed on the cell surface leading to an increase in drug concentration near the cell membrane, thus generating a concentration gradient that promotes the drug influx into the cell [16]. Besides, while part of free Dtx molecules, transported into the cytoplasm by a passive diffusion, were transported out by P-glycoprotein (P-gp) pumps, NPs are taken up by cells through an endocytosis pathway, thus resulting in a higher cellular uptake of the entrapped drug [22], thereby enabling them to escape from the effect of P-gp pumps. Moreover, intracellular delivery of Dtx NPs allows a drug accumulation near the site of action [23].

## Conclusions

In this work, poly(lactide-co-caprolactone) and poly(lactide-co-caprolactone-co-glycolide) copolymers were successfully employed for the first time for the nanoencapsulation of Dtx, by using a modified nanoprecipitation method. Among the investigated copolymers, PLGA-PCL resulted more effective than PLA-PCL-NPs to achieve NPs with small size, uniform distribution, and higher encapsulation efficiency. The *in vitro *studies proved an increased antiproliferative efficacy of PLGA-PCL-Dtx NPs compared with free Dtx, in a dose- and time-dependent manner, against PC3 cells.

In conclusion, the PLGA-PCL copolymer can be considered as alternative and promising biocompatible polymer to be used on development of NP-based drug delivery system for cancer chemotherapy.

## Competing interests

The authors declare that they have no competing interests.

## Authors' contributions

VS conceived of the study, participated in its design and coordination, carried the nanoformulation experiments, and wrote the manuscript. AMR participated in the design of the study, carried the HPLC experiments and contributed to data interpretation. AMP and AC performed the biological experiments, and GP analyzed and organized the results. SM carried out the SEM analysis. AM and VA performed the DSC measurements and organized data analysis. SU has been involved in revising the manuscript critically. MS participated in the experimental setup development as well as discussions and data analysis, and helped in writing the manuscript. All authors read and approved the final manuscript.

## References

[B1] Jemal A Siegel R Ward E Hao Y Xu J Murray T Thun MJ Cancer statistics, 2009 CA Cancer J Clin 2009 59 225 249 10.3322/caac.20006 19474385

[B2] Petrylak DP Tangen CM Hussain MH Lara PN Jr Jones JA Taplin ME Burch PA Berry D Moinpour C Kohli M Benson MC Small EJ Raghavan D Crawford ED Docetaxel and estramustine compared with mitoxantrone and prednisone for advanced refractory prostate cancer N Engl J Med 2004 351 1513 1520 10.1056/NEJMoa041318 15470214

[B3] Tannock IF de Wit R Berry WR Horti J Pluzanska A Chi KN Oudard S Théodore C James ND Turesson I Rosenthal MA Eisenberger MA TAX 327 Investigators Docetaxel plus prednisone or mitoxantrone plus prednisone for advanced prostate cancer N Engl J Med 2004 351 1502 1152 10.1056/NEJMoa040720 15470213

[B4] Petrylak DP The treatment of hormone-refractory prostate cancer: docetaxel and beyond Rev Urol 2006 8 S48 S55 17021642 PMC1578715

[B5] Lee E Kim H Lee IH Jon S In vivo antitumor effects of chitosan-conjugated docetaxel after oral administration J Control Release 2009 140 79 85 10.1016/j.jconrel.2009.08.014 19712714

[B6] Musumeci T Ventura C Giannone I Ruozi B Montenegro L Pignatello R Puglisi G PLA/PLGA nanoparticles for sustained release of docetaxel Int J Pharm 2006 325 172 179 10.1016/j.ijpharm.2006.06.023 16887303

[B7] Zhang Z Feng SS Nanoparticles of poly(lactide)/vitamin E TPGS copolymer for cancer chemotherapy: synthesis, formulation, characterization and in vitro drug release Biomaterials 2006 27 262 270 10.1016/j.biomaterials.2005.05.104 16024075

[B8] Zhang H Cui W Bei J Wang S Preparation of poly(lactide-co-glycolide-co-caprolactone) nanoparticles and their degradation behaviour in aqueous solution Polym Degrad Stab 2006 91 1929 1936 10.1016/j.polymdegradstab.2006.03.004

[B9] Hans ML Lowman AM Biodegradable nanoparticles for drug delivery and targeting Curr Opin Solid State Mater Sci 2002 6 319 327 10.1016/S1359-0286(02)00117-1

[B10] Pinto Reis C Neufeld RJ Ribeiro AJ Veiga F Nanoencapsulation I. Methods for preparation of drug-loaded polymeric nanoparticles Nanomedicine 2006 2 8 21 17292111 10.1016/j.nano.2005.12.003

[B11] Sahoo SK Panyam J Prabha S Labhasetwar V Residual polyvinyl alcohol associated with poly(D,L-lactide-co-glycolide) nanoparticles affects their physical properties and cellular uptake J Control Release 2002 82 105 114 10.1016/S0168-3659(02)00127-X 12106981

[B12] Khattak SF Bhatia SR Roberts SC Pluronic F127 as a cell encapsulation material: utilization of membrane-stabilizing agents Tissue Eng 2005 11 974 983 10.1089/ten.2005.11.974 15998236

[B13] Moghimi SM Hunter AC Poloxamers and poloxamines in nanoparticle engineering and experimental medicine Trends Biotechnol 2000 18 412 420 10.1016/S0167-7799(00)01485-2 10998507

[B14] Yan F Zhang C Zheng Y Mei L Tang L Song C Sun H Huang L The effect of poloxamer 188 on nanoparticle morphology, size, cancer cell uptake, and cytotoxicity Nanomedicine 2010 6 170 178 19447200 10.1016/j.nano.2009.05.004

[B15] Immordino ML Brusa P Arpicco S Stella B Dosio F Cattel L Preparation, characterization, cytotoxicity and pharmacokinetics of liposomes containing docetaxel J Control Release 2003 91 417 429 10.1016/S0168-3659(03)00271-2 12932719

[B16] Fonseca C Simoes S Gaspar R Paclitaxel-loaded PLGA nanoparticles: preparation, physicochemical characterization and in vitro anti-tumoral activity J Control Release 2002 83 273 286 10.1016/S0168-3659(02)00212-2 12363453

[B17] Cho K Wang X Nie S Chen Z Shin DM Therapeutic nanoparticles for drug delivery in cancer Clin Cancer Res 2008 14 1310 1316 10.1158/1078-0432.CCR-07-1441 18316549

[B18] Mehta AK Yadav KS Sawant KK Nimodipine loaded PLGA nanoparticles: formulation optimization using factorial design, characterization and in vitro evaluation Curr Drug Deliv 2007 4 185 193 10.2174/156720107781023929 17627492

[B19] Korsmeyer RW Gurney R Doelker E Buri P Peppas NA Mechanism of solute release from porous hydrophilic polymer J Pharm Sci 1983 15 25 35 10.1002/jps.2600721021 6644570

[B20] Jeong SI Kim BS Kang SW Kwon JH Lee YM Kim SH Kim YH In vivo biocompatibilty and degradation behavior of elastic poly(L-lactide-co-epsilon-caprolactone) scaffolds Biomaterials 2004 25 5939 5946 10.1016/j.biomaterials.2004.01.057 15183608

[B21] Luo Y Ling Y Guo W Pang J Liu W Fang Y Wen X Wei K Gao X Docetaxel loaded oleic acid-coated hydroxyapatite nanoparticles enhance the docetaxel-induced apoptosis through activation of caspase-2 in androgen independent prostate cancer cells J Control Release 2010 147 278 288 10.1016/j.jconrel.2010.07.108 20655966

[B22] Panyam J Labhasetwar V Dynamics of endocytosis and exocytosis of poly(D,L-lactide-co-glycolide) nanoparticles in vascular smooth muscle cells Pharm Res 2003 20 212 220 10.1023/A:1022219003551 12636159

[B23] Yoo HS Park TG Folate-receptor-targeted delivery of doxorubicin nano-aggregates stabilized by doxorubicin-PEG-folate conjugate J Control Release 2004 100 247 256 10.1016/j.jconrel.2004.08.017 15544872

